# Supplementary material for: Lipopolysaccharides derived from Porphyromonas gingivalis and Escherichia coli: Differential and interactive effects on novelty-induced hyperlocomotion, blood cytokine levels and TLR4-related processes
Source: PLoS One. 2024 Jun 10;19(6):e0292830. doi: 10.1371/journal.pone.0292830 (PMC11164397; doi:10.1371/journal.pone.0292830)
Supplement: S1 File — (PDF) [file pone.0292830.s001.pdf]

Table 1

		Body weight (g)		Spleen weight (mg)	
Without treatment		23		120.2	
		23		112.5	
		23		144.6	
		Mean	S.E.M.	Mean	S.E.M.
n =	3	23.0	0.0	125.8	9.7
Vehicle		23		143.5	
		23		156.1	
		23		110.5	
		Mean	S.E.M.	Mean	S.E.M.
n =	3	23.0	0.0	136.7	13.6
TAK-242 (3.0 mg/kg)		23		129.8	
		23		105.0	
		23		116.7	
		22		123.2	
		29		164.8	
		29		145.5	
		Mean	S.E.M.	Mean	S.E.M.
n=	6	24.8	1.3	130.8	8.7
Pg -LPS (500 µg/kg)		23		110.2	
		22		103.3	
		22		103.0	
		Mean	S.E.M.	Mean	S.E.M.
n =	3	22.3	0.3	105.5	2.4
Pg-LPS (840 µg/kg)		21		100.7	
		21		186.7	
		21		153.7	
		Mean	S.E.M.	Mean	S.E.M.
n =	3	21.0	0.0	147.0	25.1
Ec -LPS (840 µg/kg)		22		115.7	
		22		118.2	
		22		108.2	
		Mean	S.E.M.	Mean	S.E.M.
n =	3	22.0	0.0	114.0	3.0
TAK-242 (3.0 mg/kg) + *Ec* -LPS (840 µg/kg)		22		130.2	
		22		126.5	
		22		140.0	
		23		146.6	
		27		117.1	
		29		135.0	
		Mean	S.E.M.	Mean	S.E.M.
n =	6	24.2	1.2	132.6	4.2
Pg -LPS (500 µg/kg) + *Ec* -LPS (840 µg/kg)		22		117.2	
		22		137.3	
		21		126.7	
		22		178.5	
		29		167.4	
		Mean	S.E.M.	Mean	S.E.M.
n =	5	23.2	2.1	145.4	11.8

Table 2

		CD4+ T Cell (x 10*8)		CD21+ B Cell (x 10*8)	
Without treatment		4.7		31.3	
		6.6		19.9	
		4.5		22.1	
		Mean	S.E.M.	Mean	S.E.M.
n=	3	5.3	0.7	24.4	3.5
Vehicle		5.4		24.1	
		6.7		44.9	
		5.0		22.9	
		Mean	S.E.M.	Mean	S.E.M.
n=	3	5.7	0.5	30.6	7.1
TAK-242 (3.0 mg/kg)		4.3		8.4	
		5.2		11.8	
		6.2		15.5	
		8.9		27.6	
		11.6		36.1	
		Mean	S.E.M.	Mean	S.E.M.
n=	5	7.2	1.3	19.9	5.2
Pg-LPS (500 µg/kg)		8.9		12.5	
		9.2		8.2	
		6.2		12.0	
		Mean	S.E.M.	Mean	S.E.M.
n=	3	8.1	1.0	10.9	1.3
Pg-LPS (840 µg/kg)		4.4		15.5	
		4.3		21.4	
		6.6		34.0	
		Mean	S.E.M.	Mean	S.E.M.
n=	3	5.1	0.7	23.7	5.5
Ec-LPS (840 µg/kg)		5.6		25.3	
		3.4		18.7	
		4.5		21.9	
		Mean	S.E.M.	Mean	S.E.M.
n=	3	4.5	0.6	22.0	1.9
TAK-242 (3.0 mg/kg) + Ec-LPS (840 µg/kg)		14.5		36.0	
		6.9		31.8	
		8.8		27.0	
		11.2		23.6	
		7.1		20.9	
		Mean	S.E.M.	Mean	S.E.M.
n=	5	9.7	1.4	27.8	2.7
Pg-LPS (500 µg/kg) + Ec-LPS (840 µg/kg)		6.0		34.3	
		10.4		30.8	
		7.2		20.5	
		20.5		24.0	
		9.7		19.2	
		Mean	S.E.M.	Mean	S.E.M.
n=	5	10.8	2.6	25.8	2.9

Fig 1

	Vehicle	Pg-LPS 100	Pg-LPS 500	Pg-LPS 840
	3166	4545	3211	1500
	2847	1911	1086	3398
	2165	1261	1328	2217
	605	1808	2628	2670
	2593	1829	2764	1504
	2706	2351	2742	1593
n =	6	6	6	6
Mean	2347	2284	2293	2147
S.E.M.	373	474	354	315

	Vehicle	Pg-LPS 100	Pg-LPS 500	Pg-LPS 840
	1579	393	1940	282
	3866	2634	469	171
	1799	3537	1192	142
	1990	2624	333	272
	1334	1820	3577	856
	2424	2158	390	1678
	3188	2287	808	1912
			2243	
n =	7	7	8	7
Mean	2312	2207	1369	759
S.E.M.	347	364	404	283

Fig 2

	Vehicle	TAK 3	Ec-LPS 840	TAK 3/ Ec-LPS 840	Pg-LPS 500	Pg-LPS 500/ Ec-LPS 840
	1579	2345	282	4329	3252	535
	3866	3050	171	2127	3248	1989
	1799	3803	142	2196	1654	2491
	1990	2044	272	2510	1830	1830
	1334	3205	856	1259	3451	1231
	2424	2069	1678	1857	2177	3819
	3188		1912			
n =	7	6	7	6	6	6
Mean	2311.6	2752.6	759.0	2379.9	2602.1	1982.5
S.E.M.	347.0	290.6	283.4	426.0	328.4	458.8

Fig 3

Central	Vehicle	Ec-LPS
	200	7
	952	27
	102	119
	435	82
	150	141
	392	270
	865	341
n =	7	7
Mean	442.4	141.1
S.E.M.	129.1	46.7

Peripheral	Vehicle	Ec-LPS
	1531	222
	2833	141
	1665	26
	1513	190
	1153	702
	1971	1381
	2277	1533
n =	7	7
Mean	1849.1	599.3
S.E.M.	212.6	236.2

Fig 4

IL-6

	Baseline	Vehicle	Pg-LPS 840	Ec-LPS 840	TAK 3	TAK 3/ Ec-LPS 840	Pg-LPS 500	Pg-LPS 500/ Ec-LPS
	73.4	41.7	133.1	29333.0	1.5	422.0	128.0	9270.0
	3.5	49.5	23.3	3400.0	41.4	3754.0	9.4	20.8
	17.7	7.5	190.6	15956.0	26.2	12084.0	16.1	97.0
	19.3	1.3	17.9	18523.0	7.7	1908.0	48.4	22751.0
	4.5	262.7	108.7	19427.0	144.0	4569.0	2.6	
	1.5					8351.0	168.7	
n=	6	5	5	5	5	6	6	4
mean	20.0	72.6	94.7	17327.8	44.2	5181.3	62.2	8034.7
sem	11.1	48.5	33.1	4158.3	25.9	1765.1	28.5	5364.4

Fig 5

TNFalpha

	Baseline	Vehicle	Pg-LPS 840	Ec-LPS 840	TAK 3	TAK 3/ Ec-LPS 840	Pg-LPS 500	Pg-LPS 500/ Ec-LPS 840
	7.9	6.5	10.4	227.0	5.8	34.0	8.3	107.8
	6.5	6.9	7.8	45.3	5.8	113.6	1.3	6.0
	11.4	3.1	9.3	106.3	6.7	142.6	6.5	7.9
	9.0	6.0	9.5	151.1	4.5	53.5	5.5	151.0
	2.8	8.4	12.1	92.0	4.8	95.2	0.0	
	5.5					153.9	9.7	
n=	6	5	5	5	5	6	6	4
mean	7.2	6.2	9.8	124.3	5.5	98.8	5.2	68.2
sem	1.2	0.9	0.7	30.7	0.4	19.5	1.6	36.4

Fig 6

IL-10

	Baseline	Vehicle	Pg-LPS 840	Ec-LPS 840	TAK 3	TAK 3/ Ec-LPS 840	Pg-LPS 500	Pg-LPS 500/ Ec-LPS 840
	376.9	22.5	27.0	559.6	5.4	44.1	18.6	602.6
	3.0	3.0	10.4	1054.0	93.9	493.9	46.0	7.1
	86.3	0.0	4.7	2660.0	4.7	456.7	0.0	12.0
	55.4	5.4	5.4	956.9	29.7	287.5	32.9	863.9
	25.6	29.2	172.8	4014.0	0.0	530.0	3.2	
	0.0					351.5	79.2	
n=	6	5	5	5	5	6	6	4
mean	91.2	12.0	44.0	1848.9	26.7	360.6	30.0	371.4
sem	58.7	5.8	32.4	649.4	17.6	73.3	12.2	215.6
